# The Application of Rho Kinase Inhibitors in the Management of Glaucoma

**DOI:** 10.3390/ijms25115576

**Published:** 2024-05-21

**Authors:** Li-Ching Liu, Yi-Hao Chen, Da-Wen Lu

**Affiliations:** Department of Ophthalmology, Tri-Service General Hospital, National Defense Medical Center, Taipei 11490, Taiwan; ophelia30330@gmail.com (L.-C.L.); keanechen18@gmail.com (Y.-H.C.)

**Keywords:** glaucoma, rho kinase inhibitors, ROCK, intraocular pressure, neuroprotection

## Abstract

Glaucoma is a chronic neurodegenerative disease that poses a significant threat of irreversible blindness worldwide. Current treatments for glaucoma focus on reducing intraocular pressure (IOP), which is the only modifiable risk factor. Traditional anti-glaucomatous agents, including carbonic anhydrase inhibitors, beta-blockers, alpha-2 agonists, and prostaglandin analogs, work by either improving uveoscleral outflow or reducing aqueous humor production. Rho kinase (ROCK) inhibitors represent a novel class of anti-glaucomatous drugs that have emerged from bench to bedside in the past decade, offering multifunctional characteristics. Unlike conventional medications, ROCK inhibitors directly target the trabecular meshwork outflow pathway. This review aims to discuss the mechanism of ROCK inhibitors in reducing IOP, providing neuroprotection, and preventing fibrosis. We also highlight recent studies and clinical trials evaluating the efficacy and safety of ROCK inhibitors, compare them with other clinical anti-glaucomatous medications, and outline future prospects for ROCK inhibitors in glaucoma treatment.

## 1. Introduction

Glaucoma is a chronic neurodegenerative disease that is characterized by progressive damage to the retinal ganglion cells, which leads to visual field defects and accounts for irreversible blindness [1]. It is estimated that by the year 2040, approximately 111.8 million people will suffer from glaucoma [2,3]. Glaucoma is commonly classified into two categories: primary or secondary, depending on the underlying cause of the disease. Primary glaucoma can be further divided into two subtypes: primary open-angle glaucoma (POAG) and primary angle-closure glaucoma (PACG), which are distinguished by the anatomical features of the anterior chamber. While ocular hypertension (OH) may not always present, and many patients even have normal to low intraocular pressure (IOP), IOP reduction is currently the only modifiable factor proven to decrease both the risk of disease onset and its progression. Among all presently available IOP-reducing strategies, including medication, laser, and surgery, hypotensive agents act as the first-line therapies and also the most crucial maintenance treatment.

In normal ocular physiology, the aqueous humor is produced from the ciliary body and eliminated via two pathways: the trabecular meshwork and the uveoscleral pathway. The former is a so-called conventional pathway, which accounts for 60–80% of aqueous outflow [4]. The pathway comprises aqueous humor passing through the trabecular meshwork (TM) across the Schlemm’s canal and draining into collector channels, aqueous veins, and episcleral veins. The uveoscleral pathway, also known as the unconventional pathway, is an alternative route for drainage of aqueous humor and is composed of the uveal meshwork and the anterior portion of the ciliary muscle (CM). The aqueous humor enters the connective tissue present between the muscle bundles, passes through the suprachoroidal space, and eventually exits through the sclera [5]. Traditional medication for treating glaucoma aims to either reduce the production of aqueous humor or facilitate its elimination. Medications such as carbonic anhydrase inhibitors, beta-blockers, and alpha-2 agonists are used to reduce the production of aqueous humor. Another way to manage glaucoma is by increasing outflow through the unconventional pathway with drugs such as prostaglandin analogs and alpha-2 agonists. Additionally, reducing outflow resistance in the conventional pathway can be achieved partially by using muscarinic agonists or miotics such as pilocarpine and carbachol, especially for those with angle-closure glaucoma.

While most aqueous humor outflow in humans occurs through conventional pathways, 75% of the resistance to outflow is located in the trabecular meshwork (TM). This resistance is primarily found in the inner wall region, which includes the juxtacanalicular connective tissue (JCT) and the inner wall endothelium of Schlemm’s canal (SC) [6]. The TM is a complex structure with properties of endothelial cells, myofibroblasts, and macrophages. It plays a crucial role in secreting extracellular matrix (ECM) proteins and maintaining ECM remodeling and renewal, which are essential for the normal regulation of resistance [7].

In eyes with primary open-angle glaucoma (POAG), research suggests that the increased resistance to aqueous humor outflow is associated with stiffening of the trabecular meshwork (TM). This stiffening is correlated with the apoptosis of TM cells, alterations in the ECM, and reorganization of the actin cytoskeleton [8,9]. These changes in the TM resemble age-related changes but progress more rapidly in individuals with glaucoma [7,10]. Traditional anti-glaucoma agents do not target the TM. Therefore, prior to the 2000s, surgical interventions such as trabeculectomy or trabeculotomy were the only options to modify TM structure. Rho kinase (ROCK) inhibitors, a novel class of drugs approved by the American Food and Drug Administration (FDA) in 2017, act directly on TM cells to reduce intraocular pressure (IOP) [11,12]. These inhibitors influence various signaling pathways and specifically target the TM, making them promising candidates for glaucoma treatment. This review summarizes the application of ROCK inhibitors in glaucoma treatment based on fundamental and clinical research in the recent decade.

## 2. Rho/Rho Kinase Pathway in the Role of Glaucoma

The Rho family of guanosine triphosphatases (GTPases), including RhoA, RhoB, and RhoC, are small signaling proteins that belong to the Ras G-protein superfamily. These proteins can be activated by secreted cytokines such as endothelin-1, thrombin, angiotensin II, lysophosphatidic acid, and TGF-β or by integrin activation after binding with the ECM [13]. They are activated when they bind to guanosine triphosphate (GTP) and become inactive when they bind to guanosine diphosphate (GDP). The study of the ROCK pathway in glaucoma dates back to the 1990s, when Dr. Benjamin Geiger and Dr. Paul Kaufman collaborated to investigate how cell signaling pathways regulate aqueous humor outflow resistance [14,15]. Their research showed that cytoskeletally active agents like latrunculin and H7, a protein kinase inhibitor affecting Rho kinase, can significantly decrease aqueous humor outflow resistance in monkey models [16,17,18]. These findings sparked interest in the role of cell mechanics in aqueous humor outflow and the importance of Rho kinase in this process.

The Rho-associated protein kinases (ROCK) consist of two homologous serine/threonine kinase isoforms in cells: ROCK1 (also known as ROCK-I or ROKβ) and ROCK2 (also known as ROCK-II or ROKα), are effectors that bind to the active GTP-bound Rho G-proteins to become active [19]. In humans, both ROCK1 and ROCK2 are expressed in the eye but differ in other tissue distributions. ROCK1 is mainly expressed in non-neural tissues like the heart, lung, and skeletal muscle, while ROCK2 is primarily expressed in the brain [20].

### 2.1. Role of Rho/Rho Kinase in Aqueous Outflow Pathway

The conventional aqueous outflow pathway controls the outflow resistance through the contraction and relaxation of the actomyosin system in CM, TM cells, and the inner wall endothelium of SC [21]. Previous in vitro studies demonstrated that Rho and ROCKs are expressed in the cells of the outflow pathway in human and animal models [22,23]. ROCK regulates actomyosin contractility in various cells by phosphorylating its substrates.

**Figure 1:**
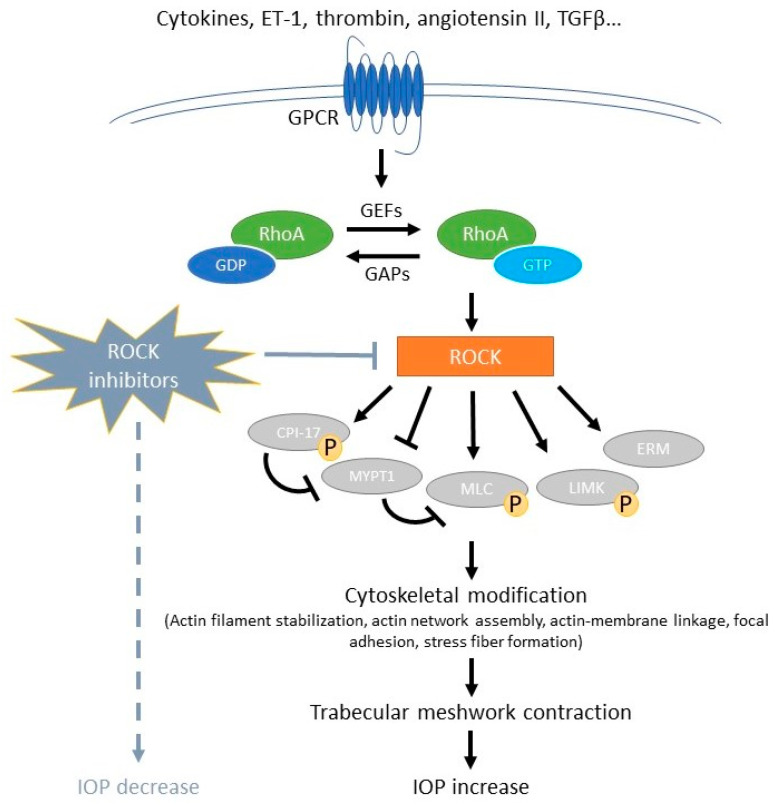
Schematic diagram of the RhoA/Rho kinase signaling pathway. When G protein-coupled receptors (GPCRs) bind to various ligands, such as endothelin-1 (ET-1), angiotensin II, and TGF-β, they activate guanine nucleotide exchange factors (GEFs) and, in turn, activate RhoA by converting it from an inactive GDP-bound state to an active GTP-bound form. This activation triggers the ROCK cascade, leading to the phosphorylation of downstream target proteins and subsequent cytoskeletal modifications. These changes cause contraction of the trabecular meshwork, reducing aqueous outflow through the conventional pathway and increasing intraocular pressure (IOP). ROCK inhibitors reduce IOP by blocking this cascade. GAP: GTPase-activating protein. ERM: ezrin-radixin-moesin. CPI-17: C-kinase-potentiated protein phosphatase one inhibitor of 17 kDa.

The cytoskeleton consists of microtubules, actin filaments (microfilaments), and intermediate filaments, which help maintain the cell’s shape, aid intracellular transport, and are crucial for cell division and motility. The activated ROCK can phosphorylate and regulate downstream proteins, such as phosphorylate myosin light chain (MLC) and myosin phosphatase substrate 1 (MYPT1), LIM (Lin11, Isl1, and Mec3) domain kinase 1 (LIMK), calponin, and ezrin-radixin-moesin proteins. Contribute to the modification of the cytoskeleton by regulation of myoglobin/actin contraction, cell morphology, polarity, proliferation, stiffness, adhesion, and matrix synthesis [14,15,24]. The activation of ROCK in TM cells promotes the phosphorylation of myosin II and enhances its contractile activity. This activation also stimulates the synthesis and assembly of ECM proteins. Conversely, the rigidity and assembly of the ECM can influence actomyosin contraction and further activate Rho GTPase. These findings suggest a possible feedback loop between ECM and Rho GTPase in TM cells [25,26,27]. Schematic diagram of the RhoA/Rho kinase signaling pathway is shown as Figure 1.

### 2.2. Role of Rho/Rho Kinase in Optic Nerve and Retina

Goldhagen et al. proved that both normal and glaucoma human eyes showed positive immunohistochemical staining for RhoA, ROCK-1, and ROCK-2 in the TM, CM, and optic nerve head (ONH), and there was a significant increase in the RhoA protein levels in the glaucomatous ONH compared to the age-matched control group [28].

In POAG, damage occurs at the ONH and is mediated by astrocytes. These cells are present in the retina, ONH, and visual brain, where they play a crucial role in balancing the activity of neurons, regulating their function, and responding to immune challenges in the retina and brain. Previous research suggests that reactive astrocytes can disrupt the homeostasis and integrity of neural and connective tissues in the ONH of both glaucoma humans and monkeys [29,30]. Lukas et al. found that cultured human glaucomatous ONH astrocytes exhibit higher RhoA GTPase and specific ECM protein activity than normal astrocytes under unstimulated conditions [31].

Research has found that ROCK can cause vasoconstriction in the arteries of the optic nerve head and can also contribute to glutamate-induced damage at the axonal level [32]. However, in adult rats with optic nerve microcrush models, the use of Rho inhibitors seems to reverse these effects, which could potentially help improve blood flow and promote the survival of retinal ganglion cells [33]. Additionally, the use of ROCK inhibitors also lead to similar positive outcomes in rabbit models [25,34]. As such, Rho/ROCK inhibitors may benefit glaucoma patients through non-IOP-dependent mechanisms, such as neuroprotection, by improving blood flow to the optic nerve and increasing ganglion cell survival [14,20]. However, most of the hypotheses regarding neuroprotection and improved blood flow are based on findings from animal models. Further studies are needed to validate these hypotheses and ascertain the clinical benefits in humans.

### 2.3. Role of Rho/Rho Kinase in Tenon Fibroblast

Traditional glaucoma surgeries, like trabeculectomy and tube shunt surgery, reduce IOP by establishing a direct route for aqueous humor to drain from the anterior chamber to the subconjunctival space. The primary reason for the postoperative failure of filtration surgery is scarring in the filtering bleb during the wound-healing process. Antimetabolites, such as mitomycin C and 5-fluorouracil, have been administered to prevent bleb fibrosis and scarring for many years. However, these agents may also result in severe side effects or complications [35].

Previous in vitro studies on human Tenon’s fibroblasts and conjunctival fibroblasts revealed that TGF-β treatment activated a series of rho-mediated cellular changes. These changes encompassed cell contraction, proliferation, adhesion, migratory response, cytoskeletal alterations, and myofibroblast transdifferentiation, marked by an increase in α-smooth muscle actin expression. The effects of TGF-β were suppressed by the administration of ROCK inhibitors such as Y-27632 [36] and ripasudil (K-115) [37]. The in vivo animal model studies also revealed similar findings. They suggested that ROCK inhibitors have therapeutic potential in reducing scar formation after glaucoma filtration surgeries by inhibiting the transdifferentiation of fibroblasts into myofibroblasts and extracellular matrix deposition in rabbit and rat models [20,38,39,40]. In real-world clinical practice, further research is needed to determine if ROCK inhibitors exhibit similar anti-fibrosis properties.

## 3. Rho Kinase Inhibitors

ROCK inhibitors play various roles, primarily by modulating the RhoA/ROCK signaling pathway. The first ROCK inhibitor, fasudil, was developed in the 1990s in Japan for treating cerebral vasospasm after subarachnoid hemorrhage due to its ability to relax smooth muscle cells [41]. This marked the beginning of research into the therapeutic potential of ROCK inhibitors in cardiovascular, neurological, and ophthalmic conditions [42]. ROCK inhibitors also have anti-inflammatory effects, inhibiting the activation of inflammatory cells and the production of inflammatory cytokines, which may be beneficial in treating asthma and inflammatory bowel disease [43,44]. Additionally, increased Rho/ROCK activity and gene expression have been observed in various cancers, leading to the development of ROCK inhibitors for potential use in cancer therapy [45].

Currently, marketed ROCK inhibitors approved for glaucoma treatment are Ripasudil (K-115; brand name: Glanatec) and netarsudil (AR-13324; brand name: Rhopressa), which target both ROCK1 and ROCK2. Many other ROCK inhibitors are under different stages of clinical trials and bench studies, mostly targeting both ROCK1 and ROCK2, such as Y-27632, H-1152, Wf-536, AMA-0076, GSK269962A, SB-772077-B, SAR407899, AR-12286, sovesudil, and RKI-1447. Some inhibitors, like SNJ-1656, ITRI-E-212, and KD-025, show selectivity for ROCK2 inhibition [15,46,47,48]. The following paragraph will introduce several recent studies focusing on ROCK inhibitors for glaucoma treatment.

## 4. Recent Trials of Rho Kinase Inhibitors in Glaucoma Treatment

### 4.1. Ripasudil (K-115)

Ripasudil (K-115, Glanatec^®^; Kowa Company, Ltd., Nagoya, Aichi, Japan) is a selective ROCK inhibitor derived from fasudil. It was the first ROCK inhibitor approved for the treatment of ocular hypertension (OH) and glaucoma in Japan in 2014. Tanihara et al. conducted a series of clinical trials, including phase I, II, and III, starting in 2013 to evaluate ripasudil’s efficacy and safety [49]. The phase II dose–response study involved 210 patients with OH or POAG with a baseline IOP around 23.0–23.4 mmHg. The study showed that the IOP significantly decreased by -3.1 mmHg at 8 h after instillation with a 0.4% concentration of ripasudil. This finding supported the use of ripasudil 0.4% with a twice-daily dosing regimen as the optimal dose for clinical treatment [50].

A 12-month post-marketing surveillance found no new safety concerns with significant IOP reductions in all baseline IOP categories and glaucoma types, except neovascular glaucoma. Ripasudil was deemed safe and effective for glaucoma and OH [51,52]. Two multicenter parallel group comparison studies of ripasudil-timolol and ripasudil-latanoprost showed significant additional IOP-lowering effects compared to placebo [53]. A retrospective cohort study by Inoue et al. demonstrated that the addition of ripasudil to existing glaucoma treatment regimens is safe and effective in reducing IOP, regardless of the number of medications in use [54].

The ripasudil-brimonidine fixed-dose combination (RBFC; also known as K-232) is the first treatment to combine a ROCK inhibitor and an α2-adrenoceptor agonist. It has demonstrated IOP-lowering ability and lower conjunctival hyperemia scores compared to single ripasudil administration [55,56]. A recent prospective phase 3 study of RBFC confirmed its long-term (52 weeks) efficacy and safety, both alone and in combination with other anti-glaucoma agents [57]. An in vivo study was conducted to treat adult mice with optic nerve injury using topical ripasudil, brimonidine, or a combination of both drugs. The study found that topical ripasudil suppressed expression of TNFα, IL-1β, and monocyte chemotactic protein-1, thus accounting for RGC rescue after optic nerve injury, and also proved that the combination of both drugs was more effective than a single treatment with either ripasudil or brimonidine [58]. However, it is important to note that these findings are limited to animal models, where the dosage of ripasudil or brimonidine differs from that used in clinical trials for patients with ocular hypertension.

Futakuchi et al. conducted a multicenter historical cohort study to evaluate the IOP-lowering effect in uveitic glaucoma (UG), exfoliation glaucoma (EG), and steroid-induced glaucoma (SG). The study enrolled 332 eyes and found that the IOP decreased statistically significantly in all three groups but was more remarkable in UG and SG than EG, which the author thought to be related to higher baseline IOP levels in UG and SG. The finding suggested that ripasudil is safe and effective in IOP-lowering for secondary glaucoma [59,60]. A recent small-scale study that enrolled 16 patients also found that post-trabeculectomy use of ripasudil on UG patients could significantly reduce IOP and lower the need for bleb revision and suture lysis [61]. Larger-scale studies are necessary to bolster this finding and assess its clinical utility. The long-term monitoring of ripasudil’s adverse drug reactions showed that the most common side effects were blepharitis (8.6%), conjunctival hyperemia (8.5%), and conjunctivitis (6.3%). Blepharitis was the main reason for discontinuing ripasudil treatment, with risk factors including female gender, previous blepharitis history, or allergy to other glaucoma medications [62,63].

### 4.2. Netarsudil (AR-13324)

Netarsudil (AR-13324, Rhopressa^®^, Aerie Pharmaceuticals, Bedminster, NJ, USA) is a ROCK and norepinephrine transporter (NET) inhibitor. In 2017, the Food and Drug Administration (FDA) in the USA approved it as a 0.02% formulation with a once-a-day dosing frequency for OAG and OH. In November 2019, it was further approved by the European Medicines Agency under the trade name Rhokiinsa for POAG and OH [64]. In both rabbit and monkey studies, AR-13324 produced large reductions in IOP (20–25%) with a longer duration. AR-13324 reduces IOP by relaxing the trabecular meshwork, increasing the outflow of aqueous humor, reducing scleral venous pressure, and directly decreasing IOP [65,66]. Further human clinical trials demonstrated the efficacy of netarsudil in IOP lowering without severe safety issues, thus leading to the approval of these new drugs [67,68,69].

The series ROCKET trials were pivotal randomized phase 3 clinical trials conducted to evaluate the efficacy (ROCKET-1[NCT02207491], ROCKET-2[NCT02207621], and ROCKET-4[NCT02558374]) and safety (ROCKET-1, ROCKET-2, ROCKET-3[NCT02246764], and ROCKET-4) of netarsudil 0.02% (Rhopressa) for the treatment of OAG and OH patients with baseline IOP < 25 mmHg by comparing once-daily netarsudil 0.02% with twice-daily timolol maleate 0.5% to demonstrate the non-inferiority [64]. The most common ocular side effect was conjunctival hyperemia (netarsudil, 54.4%; timolol, 10.4%) and mainly was graded as mild degree (77.6%) of affected netarsudil-treated patients. Other adverse events (AEs) included conjunctival hemorrhage and corneal verticillata, which were mostly self-limited and could be resolved after stopping the medication.

Freiberg et al. concluded that in people diagnosed with OH or OAG, netarsudil may be inferior to latanoprost and slightly inferior to timolol in IOP reduction, but the combination showed better efficacy [70]. The MERCURY-1 (NCT02558400) and MERCURY-2 (NCT02674854) trials are phase 3 clinical trials comparing the fixed-dose combination of netarsudil 0.02% and latanoprost 0.005% (netarsudil/latanoprost FDC, Rocklatan^®^, PG324) to netarsudil or latanoprost monotherapy in patients with OAG or OH [71,72]. Patients with unmedicated baseline IOP > 20 and <36 mmHg were randomized to receive once-daily treatment with netarsudil/latanoprost FDC, netarsudil 0.02%, or latanoprost 0.005% for up to 12 months. At the 3-month follow-up, the proportion of patients achieving at least a 40% reduction from baseline IOP was significantly higher in the netarsudil/latanoprost FDC group (30.9%) compared to the netarsudil (5.9%) and latanoprost (8.5%) groups (*p* < 0.0001 for both comparisons). The fixed-dose combination was superior to monotherapy in reducing IOP. No treatment-related serious adverse events were reported in any treatment arm, and the occurrence of conjunctival hyperemia was similar across all groups [73]. A recently published MERCURY-3 (NCT03284853) trial demonstrated that once-daily netarsudil 0.02%/latanoprost 0.005% ophthalmic solution (NET/LAT; Roclanda^®^) was non-inferior to bimatoprost 0.03%/timolol maleate 0.5% (BIM/TIM; Ganfort^®^) in IOP reduction in OAG and OH patients [74].

### 4.3. SNJ-1656 (Y-39983)

SNJ-1656 (previously Y-39983, Senju Pharmaceuticals, Osaka, Japan) was the first selective ROCK inhibitor to undergo clinical trials for reducing intraocular pressure (IOP). It is significantly more potent in inhibiting ROCK activity than Y-27632 and has shown similar IOP-lowering effects at lower concentrations. In a 2008 phase I study by Tanihara et al., SNJ-1656 demonstrated a statistically significant reduction in IOP at 4 h post-instillation with 0.05% (−1.95 mmHg, *p* < 0.001) and 0.1% (−3.00 mmHg, *p* < 0.001) concentrations compared to placebo. The main adverse effect was conjunctival hyperemia [75]. A subsequent phase II trial in 2015 confirmed its efficacy in reducing IOP in patients with OAG or OH. Adverse reactions included headache, abdominal pain, liver dysfunction, and conjunctivitis [76]. Despite no further clinical studies, SNJ-1656 remains an important agent in preclinical research for potential future applications.

### 4.4. AR-12286

AR-12286 (Aerie Pharmaceuticals, Inc., Durham, NC, USA) is a highly selective ROCK inhibitor. The Phase I and Phase II clinical trials found that AR-12286 ophthalmic solution could produce a 3 to 7 mmHg (*p* < 0.0001) reduction in IOP from baseline in normotensive subjects and 4.4 to 6.8 mmHg in OH and glaucoma patients. There was low systemic absorption, and no severe adverse events were observed [77,78]. However, there has been no further development of clinical use of this drug due to its short duration since 2017. Recent research found that AR-12286 could reverse steroid-induced morphological changes in the TM and reduce IOP in steroid-induced ocular hypertension (SIOH) eyes in mouse models that may offer insight into ROCK inhibitors in the clinical treatment of SIOH [79].

### 4.5. Sovesudil (PHP-201, AMA0076)

Sovesudil (PHP-201, formerly known as AMA0076) is a novel ROCK inhibitor designed as a locally acting soft drug. This means that after exerting its effects, the active compound is metabolized into an inactive form, reducing side effects or toxicity. Sovesudil targets the TM, where excess sovesudil is hydrolyzed by esterases into an inactive salt called AMA0078, which can be easily eliminated via tears. An animal study showed that sovesudil effectively lowers IOP in ocular normotensive and hypertensive rabbits with less hyperemia compared to treatment with latanoprost and bimatoprost [80]. A phase II study in patients with normal tension glaucoma (unmedicated baseline IOP ≤ 21 mmHg) showed that 0.25% and 0.5% concentrations of sovesudil administered three times daily significantly reduced IOP, with the 0.5% concentration demonstrating superiority over placebo. A relatively low incidence of conjunctival hyperemia was also observed [48].

### 4.6. Fasudil (HA-1077)

Fasudil (HA-1077; AT877) hydrochloride is a selective ROCK inhibitor, potent calcium channel antagonist, and vasodilator. Initially developed and approved in Japan in the 1990s for treating cerebral vasospasm after subarachnoid hemorrhage, it later demonstrated a beneficial effect on end-stage POAG patients by effectively lowering IOP [81]. However, its hydrophilic nature limits its ocular permeation and bioavailability, hindering its widespread use in glaucoma treatment. Recent studies have focused on novel drug delivery systems to enhance bioavailability, such as loading fasudil into poly (lactide-co-glycolide) (PLGA) microspheres, liposomal thermosensitive in situ gel, and chitosan nanoparticles, which could improve its effectiveness and prolong its duration of action [82,83,84].

### 4.7. Other ROCK Inhibitors in Preclinical Studies or under Clinical Trials

Several new ROCK inhibitor ophthalmic solutions are currently in clinical trials, but their results are limited and not widely available. The phase I/II study of H-1337 ophthalmic solution demonstrated clinically and statistically significant ocular hypotensive activity and was well tolerated, with a relatively low incidence of hyperemia [85]. In a phase II study of VVN539, both 0.02% and 0.04% concentrations showed statistically significant reductions in IOP compared to the vehicle group in patients with POAG and OH [86]. Further research is needed to evaluate the clinical utility of both compounds fully.

ROCK inhibitors under clinical trials in the glaucoma treatment field continue to develop, but some have not yet produced available results (Table 1). Other ROCK inhibitors are still under preclinical studies. For example, ITRI-E-212, a novel and highly specific ROCK2 inhibitor, effectively reduced IOP in normotensive and ocular hypertensive New Zealand White rabbit models and was sustained for at least 6 h after each once-daily dose [47]. The ongoing studies on ROCK inhibitors provide promising possibilities for treating glaucoma.

## 5. ROCK Inhibitors in Other Ocular Diseases

ROCK inhibitors are used not only in managing glaucoma but also in treating various other ocular diseases. In a study conducted by Okumura et al. in 2009, it was found that the selective ROCK inhibitor, Y-27632, enhanced the proliferation of corneal endothelial cells (CECs), promoted their adhesion onto a substrate, and suppressed CEC apoptosis [87]. The researchers further demonstrated that ROCK inhibitors could facilitate corneal endothelial wound healing in rabbit and primate models, as well as in four human clinical case series [88,89]. Topical treatment with ROCK inhibitors has shown potential in the management of Fuchs endothelial corneal dystrophy (FECD), corneal edema resulting from acute surgical trauma and other causes, and in tissue engineering therapy for endothelial diseases. Nevertheless, the absence of multicenter clinical trials limits the availability of robust evidence supporting the use of ROCK inhibitor eye drops in patients with FECD or other corneal endothelial disorders [90].

ROCK inhibitors also play a significant role in the management of diabetic retinopathy (DR). The ROCK signaling pathway upregulates vascular endothelial growth factors (VEGFs), which are pivotal in the pathogenesis of microvascular complications such as increased retinal vessel permeability, macular edema, vascular occlusion, and retinal ischemia in diabetic patients [91]. Over the past decade, numerous in vitro and in vivo studies have explored the effects of ROCK pathway inhibition on DR and diabetic macular edema (DME), consistently yielding positive outcomes [91,92]. However, there is still some debate regarding the optimal route of administration, the most effective dosage, and the lack of level-one evidence supporting their use in clinical treatment. More and more studies are exploring this promising field to uncover new therapeutic opportunities.

## 6. Drug Delivery System of Rho Kinase Inhibitors

Currently, FDA-approved ROCK inhibitors for glaucoma treatment include the topical ophthalmic solutions Ripasudil and Netarsudil. While topical formulations are the most common and convenient method of delivering ROCK inhibitors to the eye, they face challenges related to permeability and bioavailability, limiting their effectiveness in treating posterior segment ocular diseases. The retention of instillation sites may also lead to adverse effects on the ocular surface. To address these challenges, nanoparticles such as PLGA microspheres and chitosan nanoparticles have been designed to encapsulate ROCK inhibitors, providing better drug stability, targeted delivery, and controlled release [82,83,84]. Intravitreal injections offer a more direct route to treat posterior ocular diseases. Netarsudil, with its more lipophilic physicochemical and pharmacological properties, is a suitable candidate for intravitreal administration. AR-13503, a prodrug of netarsudil, has been investigated as a biodegradable sustained-release intravitreal implant in animal models [93], with ongoing clinical trials focusing on DME and neovascular age-related macular degeneration patients (NCT03835884). Other delivery systems have been proposed in animal studies, such as drug-loaded thin polymeric films for corneal endothelial dysfunction [94] and Y27632-PLGA-modified intraocular lenses, which can prevent posterior capsular opacification formation [95]. Overall, these drug delivery systems aim to enhance the efficacy and safety of ROCK inhibitors for treating various ocular diseases. However, further studies are needed to better understand their clinical utility.

## 7. Conclusions and Future Perspectives

ROCK inhibitors represent a novel class of medications with multifunctional characteristics for treating glaucoma and ocular hypertension, showing promising advancements from laboratory research to clinical application. The novelty in ROCK inhibitors lies in their direct targeting of the ciliary muscle, trabecular meshwork cells, and Schlemm’s canal to reduce aqueous humor production and increase conventional aqueous outflow pathway while also regulating cytoskeletal remodeling to reduce stiffness. Currently, approved ROCK inhibitors in the market include ripasudil (Glanatec^®^), netarsudil (Rhopressa^®^), and the fixed combination of netarsudil/latanoprost (Rocklatan^®^, Roclanda^®^). They have all been proven to significantly reduce IOP with acceptable side effects, primarily mild to moderate conjunctival hyperemia. While monotherapy with ROCK inhibitors may not definitively show superiority over other conventional anti-glaucomatic agents, the combination of netarsudil and latanoprost has demonstrated additional IOP reduction compared to single use. Additionally, combined ripasudil and timolol also lead to better IOP reduction efficacy than timolol monotherapy [70].

Another newly developed anti-glaucoma agent is latanoprostene bunod 0.024% (LBN, Vyzulta^®^), a nitric oxide-donating prostanoid indicated for reducing IOP in patients with OAG or OHT. Several clinical trials have demonstrated the superiority of LBN over monotherapy with timolol and latanoprost. Although no direct comparisons are available between LBN and ROCK inhibitors, both have shown efficacy in additional lowering of IOP, which does not depend on the number of baseline IOP-lowering medications [96]. A study comparing netarsudil and LBN as adjunctive therapy to maximum medical treatment in POAG patients indicated that the addition of netarsudil was associated with a significantly greater magnitude of IOP reduction (>10% reaching the therapeutic threshold) than exchanging pre-existing prostaglandins with LBN [97]. However, these studies are primarily retrospective cohort studies that need further multicenter randomized controlled trials to provide better clinical evidence. The thriving development of new anti-glaucoma agents provides more opportunities for better IOP control despite using maximum conventional medication and may potentially delay the need for surgery.

To date, glaucoma has been considered a neurodegenerative disease, and normal tension glaucoma represents a significant proportion of cases. The range and degree of IOP reduction may differ from those with ocular hypertension; therefore, functional and structural analyses such as visual field and retinal nerve fiber layer thickness may become important indicators in disease progression follow-up. ROCK inhibitors, which are hypothesized to provide more than just IOP reduction but also neuroprotection of retinal ganglion cells and antifibrotic characteristics, may offer additional benefits in glaucoma treatment. However, previous studies on the neuroprotective and antifibrotic effects of ROCK inhibitors mainly relied on in vitro or animal models, lacking clinical quantification methods to assess their efficacy in humans. Further research is necessary to comprehensively understand the advantages and disadvantages of ROCK inhibitors in real-world glaucoma treatment and to characterize their neuroprotective and antifibrotic properties in human eyes.

Lastly, a commonly reported side effect of ROCK inhibitors is conjunctival hyperemia, which can be explained by the vasodilation effect. Some other side effects that might cause more serious clinical influences include corneal verticillata, reticular epithelial corneal edema, and blepharitis. While systemic effects have been discussed in previous studies and revealed to carry low risks, vulnerable groups such as pregnant women and children raise significant concerns, as no trials currently include these groups. Most adverse reactions to ROCK inhibitors are considered acceptable and reversible upon medication discontinuation. Ongoing advancements in pharmacological drug delivery technologies may further enhance bioavailability and reduce side effects, promising a bright future in the development of ROCK inhibitors.

## Figures and Tables

**Table 1:** Other registered clinical trials of ROCK inhibitors.

Intervention Compound	Phase/Trial ID	Conditions	Year
INS115644	Phase 1/NCT00443924	POAG and OH	2007
INS117548	Phase 1/NCT00767793	Bilateral OH or Early POAG	2008
LX7101	Phase 1 and 2/NCT01528111	POAG and OH	2012
ATS907	Phase 1/2a/NCT01520116	POAG and OH	2012
	Phase 2/NCT01668524	POAG and OH	2012
H-1337	Phase 2/NCT05913232	POAG and OH	2023

## Data Availability

Not applicable.
